# Policies enacted during COVID-19 came with unintended health benefits: why go back?

**DOI:** 10.1186/s12913-023-09448-x

**Published:** 2023-05-16

**Authors:** Linda Sprague Martinez, Judith C. Scott, Melanie Rocco, Serena Rajabiun, Cecilia Flores Rodriguez, Ramona Cummings, Erin McKinney-Prupis, Malika Minott, Joy Walker-Jones, Alicia Downes, Angela Wangari Walter

**Affiliations:** 1 Boston University School of Social Work, Boston, MA US; 2 University of Massachusetts Lowell, Lowell, MA US; 3 The Alliance for Positive Change, New York, NY US; 4 University of California San Francisco, San Francisco, CA US; 5 AIDS United, Washington, DC US

**Keywords:** COVID-19, Black women with HIV, Racial capitalism, Policies that promote health

## Abstract

**Objectives:**

To explore the impact of COVID-19 on the implementation of bundled interventions to improve the engagement and retention of Black women in HIV care.

**Methods:**

Pre-implementation interviews conducted between January and April 202 L with 12 demonstration sites implementing bundled interventions for Black women with HIV. Directed content analysis was employed to examine the site interview transcripts.

**Results:**

The pandemic intensified barriers to care and harmful social conditions. However, COVID-19 also forced pivots in health care and social service delivery and some of these changes benefited Black women living with HIV.

**Conclusions:**

The continuation of policies that support the material needs of Black women with HIV and ease access to care is critical. Racial capitalism impedes the enactment of these policies and thus threatens public health.

Racial inequities in coronavirus disease 2019 (COVID-19) have been well documented [1]. Not by chance, Black people have been disproportionately impacted by COVID-19 morbidity, hospitalizations, and mortality [2]. Black women, in particular, were significantly more likely to die from COVID-19 than white men and women [3]. COVID-19 has amplified the health impacts of our racially stratified society, [4] casting a light on the ways in which Black women are left exposed harmful conditions as the result of their position in the labor market [5–8]. Black women occupy frontline positions such as nursing assistant, cashier, customer service representative, nurse and personal care aid and teacher; [9] throughout the pandemic these professions consistently experienced shortages in personal protective equipment (PPE). Women in these professions served the public and cared for their families while navigating a high likelihood of COVID-19 exposure and state sponsored violence against Black women [10].

For Black women, disproportionate disease burden and impact are not unique to COVID-19. Black women experience excess morbidity and mortality across a spectrum of chronic conditions, [11] including HIV [12]. Black women face significantly higher rates of new HIV infection than all other women, [13] are less likely to be virally suppressed, [14] and more likely to experience premature mortality [15]. A review of barriers experienced by Black women with HIV identified structural determinants associated with treatment, which included social needs as well as inequities in health care delivery [16]. Moreover, Black Women with HIV face intersecting forms of oppression based on race, gender and class coupled with the various forms of stigma (e.g., social, comorbid disease related mental health, substance use, etc.) associated with HIV, which persist despite efforts to reduce them.

Chinn and colleagues (2021) describe the ways in which race, gender, and class converge under racial capitalism to produce health inequity, while also highlighting the resilience of Black women, the need for interventions that reflect their priorities, and the socio-political conditions that shape their health and overall life chances [11]. Racial capitalism, “a specific form of capitalism, which developed in the United States as a result of Europeans’ and later white Americans’ evolving relationships with different peoples of color”, [17] creates the conditions that produce poor health among Black women [18]. Under racial capitalism, Black people are commodified, which can contribute to disproportionate levels of disease exposure [19]. This has been particularly salient for Black women, as patriarchy intersects with racial capitalism contributing to multiple forms of dehumanizing oppression including exploitation, violence, and marginalization, [18] which includes both a history of medical experimentation, [20] and present-day medical neglect by the health care system [21].

To address pervasive racial inequity experienced by Black Women with HIV, the Health Resources and Services Administration’s (HRSA) HIV/AIDS Bureau (HAB) Ryan White HIV/AIDS Program (RWHAP) Part F Special Projects of National Significance (SPNS) launched the Improving Care and Treatment Coordination: Focusing on Black Women with HIV initiative. The four-year initiative, referred to as Black Women First (BWF) began in September 2020 during the midst of the COVID-19 pandemic, at a time when disruption in essential services was intensifying. Health care was moving to virtual platforms and both elective and non-elective procedures were being cancelled. Access to care and treatment for chronic conditions such as HIV needed to be reimagined, especially for low-income communities of color as resources were shifted to attend to the soaring hospitalization rates caused by COVID-19. Layoffs and closures were rampant due to decrease in patient volume and revenue [22]. Resource shortages were found to contribute to low staff morale, and disruption to patient-centered service provision [23]. Access to health and social care services decreased and the need for social determinants of health such as housing, employment, health care services intensified.

This paper draws on data from the pre-implementation phase of the BWF to explore the ways in which the COVID-19 pandemic intensified barriers to care and harmful social conditions for Black women with HIV, as well as how changes in government and organizational policies related to COVID-19 allowed for organizations to adapt and innovate in ways that made service provision more meaningful and expansive for Black women with HIV. The findings are discussed in the context of the literature and the question of why it took a pandemic to catalyze systems change is contemplated. Finally, recommendations for improving the health and well-being of Black women with HIV moving forward are presented.

## Methods

The study was approved by the University of Massachusetts, Lowell and the Boston University Charles River Campus Institutional Review Boards (IRB), protocol numbers 20–147 and 5832X. To explore themes in the data related to COVID-19, data from pre-implementation interviews conducted with demonstration sites as they prepared to implement bundled evidence-based/informed interventions was examined. Site interviews were conducted between January and April 202 L. Informed consent was administered by Evaluation and Technical Assistance Provider (ETAP) staff prior to the site team interview.

### Demonstration Sites

**Fig. 1:**
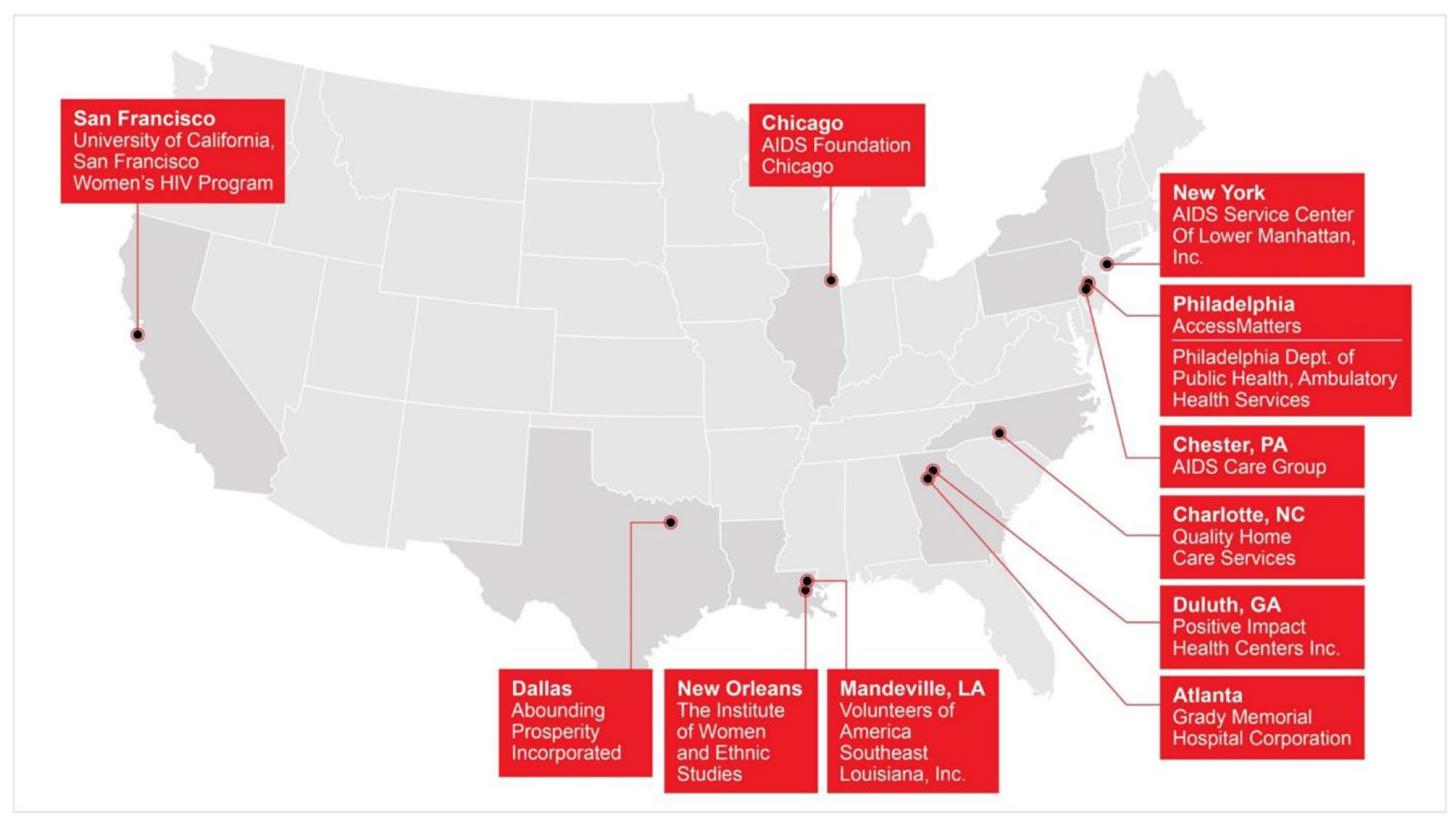
HRSA SPNS Black Women First Initiative Demonstration Sites Map

Demonstration sites (n = 12) included, AIDS service organizations, public health departments and health centers. Sites, which are situated across the country (see: Fig. 1), funded to implement bundled evidence–informed interventions for Black women with HIV to address the socio-cultural determinants of health, expand the delivery and utilization of comprehensive HIV care and treatment services, support the continuous engagement in care, and improve health outcomes for Black women with HIV in a culturally sensitive and responsive manner [24].

Intervention in the bundles include enhanced patient navigation, case management or peer engagement, Red Carpet Care experience to address barriers to HIV care, stigma reduction interventions, use of Trauma Informed Care (TIC) interventions, self-efficacy, health literacy and resiliency interventions and interventions to address Intimate Partner Violence (IPV), sexual violence or other behavioral needs [24].

### Interview procedures

Interviews were conducted with site implementation teams, which included agency leaders, evaluators, and implementation staff and well as implementation partners. Interviews were scheduled by ETAP staff during the monthly site coaching call. Teams were provided with an electronic copy of the script prior to the pre-implementation interview. The overall pre-implementation interview script explored implementation strategies sites use to support the adoption and replication of evidence informed bundled interventions as well as the ways in which COVID-19 was impacting implementation and COVID-19 related adaptations sites were making to their bundled (a copy of the interview protocol is available upon request). During the interview a member of the ETAP read through the items in the script, facilitating a discussion with the team. Interviews lasted 90–120 min and were conducted over two meeting sessions. All interviews were conducted, and audio recorded via the Zoom videoconferencing platform.

### Data management and analysis

Research staff cleaned Zoom-generated transcripts by comparing them with the audio recordings and correcting inaccuracies in the text. Once cleaned, transcripts were managed using NVivo 12.0 qualitative data management software [25]. The data were analyzed using directed content analysis [26]. Data were coded specifically related to impact of COVID-19 pandemic on the organization and staff. Two members of the research team coded all transcripts. This process involved selecting and assigning text to content codes in NVivo 12.0. Memo writing was used by coders when they encountered relevant text that did not fit the coding criteria or in cases where there were questions regarding the text.

Coder NVivo files were merged once all data was coded to determine intercoder reliability and identify areas of discrepancy. Cases were discussed when the Cohen’s kappa co-efficient was low (below 0.4) [27] and percent agreement was below 80% the coding was revisited. The coders resolved discrepancies through discussions on Zoom. After coding was completed, reports were generated for each code and summaries were prepared. This manuscript draws on data from codes focused on the impact of COVID-19. The summaries were discussed by members of the evaluation team to identify the larger narrative within the data. In the final phase of analysis, illustrative quotes from transcripts were selected to reflect a succinct, cogent written story of the data within and across the identified themes from the data.

## Findings

Implementation teams from all twelve demonstration sites participated in initial site interviews. Participants included administrative and financial leadership, project directors, intervention staff (peers, navigators, managers, providers) and evaluators as well as implementation partners. The number of team members ranged from 6 to 16. A list of participant roles by cite can be seen in Table 1.

**Table 1:** Attendance and roles by site approximately

BWF Demonstration Site	Type of Organization	Administrative or Fiscal Leadership	Project Directors	Intervention Staff	Evaluation Staff	Total
Abounding Prosperity	HIV Primary Care Clinic	0	1	4	2	7
Access Matters	Community Based Health Organization	6	2	7	1	16
AIDS Care Group	AIDS Service Organization	2	1	3	1	7
AIDS Foundation Chicago	AIDS Service Organization	5	2	3	5	15
AIDS Service Center of Lower Manhattan, Inc.	AIDS Service Organization	4	2	6	2	14
City of Philadelphia Dept. of Public Health	City Health Department	0	1	6	2	9
Grady Memorial Hospital	Public Hospital	3	0	2	1	6
The Institute of Women and Ethnic Studies	Community Based Health Organization	4	2	8	1	15
Positive Impact Health Centers Inc.	HIV Primary Care Clinic	1	1	5	1	8
Quality Home Care Services	Community Based Health Organization	2	1	4	2	9
UCSF Women’s HIV Program	HIV Primary Care Clinic	0	2	5	2	9
VOASELA	Community Based Health Organization	2	1	3	2	8

Multiple themes emerged during pre-implementation interviews. Sites described the impacts of COVID-19 as amplifying existing harmful conditions experienced by Black women including housing insecurity and barriers to care, as well as the lack of affordable childcare. Sites, meanwhile, focused on how the pandemic increased pre-existing challenges engaging Black women in care. They also describe changes made at the organizational level to support Black women with HIV. Of note site teams reported that in some cases, federal and state level expenditures and rule changes associated with pandemic relief increased access to care for Black women, in addition to addressing material need.

### Impacts on black women with HIV

Sites described the impact of COVID-19 on treatment seeking and exacerbating social need. Treatment-seeking barriers included clinical closures and patient cancellations due to fears of using public transit. However, there was a sense among sites that social need during the pandemic did not present new challenges, more so deleterious social circumstances resulting from racial capitalism were further exacerbated by COVID-19. One site noted, …*these are the same issues that was around before COVID, it just got worse*. Another, meanwhile, discussed the challenges of economic stability that were further complicated by the pandemic, *…and economic let’s not forget economic challenges. I mean, these are always [present] for many [minoritized] women living at the edges in terms of their financial stability*.

Sites reported the pandemic amplified the conditions of life under racial capitalism which produces inequitable living conditions.

*…[things are] further exacerbated conditions due to COVID …in our proposal, we talked a lot about the fractured systems that exist in our community and those systems that our population interacts with …, the complexities and the intersections create conditions that further exacerbate poor health for our particular population [Black women] and none of that has changed. I think if anything, you know, we see just a continuation of poor health outcomes, a further fracturing of systems, ongoing systemic racism and inequities within our community that further create these conditions in …a heavy burden on the population we’re working with COVID intersections…*

Housing security was an issue prior to the pandemic. Demonstration sites discussed how pandemic related job loss further fueled housing security during COVID-19.

*…there’s all these levels, housing has become more unstable, especially when people’s jobs are at risk with the service industry being somewhat decimated in New Orleans … that lack of [housing]stability adds another layer on top of everything.*

Under racial capitalism workers have few rights and little protection. The stress associated with being a frontline worker was elevated for Black Women with HIV. Sites described the burden of risk and the stress of navigating the frontlines with HIV.

.*…increase health risk especially with so many of the women being essential workers and frontline workers and being further exposed …. while living with HIV. I think the health risks involved create another level of anxiety and stress and exposure.*

In the US, the lack of affordable childcare was a burden before COVID-19. Sites discussed added challenges associated with COVID-19 and caregiving. They described how socioeconomic stressors in addition to the uncertainty of parenting during COVID-19 impacted self-care for women.

*… when a woman is stressing about her apartment, and she has kids to raise she’s not thinking about going to the doctor. She’s not thinking …am I taking my medication at the same time every day the way I have to [take them].*

*…instability of school knowing …that the children that they’re responsible for can go to school during the day to a safe place and get a meal. To try to navigate all of that on top of taking care of themselves, you know, making all their appointments and making sure they have all their medications, … trying to balance jobs…. That’s just a lot, which just further exacerbates the mental health outcomes.*

Sites described challenges associated with the uncertainty of schooling. Closures, low confidence in the ability of schools to implement COVID-19 protocols and the risk of transmission were all concerns for women. School uncertainty impacted them and their well-being.

*…in general, COVID-19 sucks. I can’t do anything. I can’t go anywhere. I’m stuck in a house with you know someone who is not very nice. I’m stuck in the house with my kids. I can’t do [anything]. I can’t go to work, like all of those things are relevant and definitely probably impact how they are obtaining and having self-care for their diagnosis…*

### Impacts on service providers

COVID-19 catalyzed changes in organizational policies governing service delivery. The level of outreach needed, coupled with the increased need among existing clients placed an added burden on staff who were already stretched thin, working to provide services to diverse Black women. In some cases, the closure of other community organizations meant that their staff on the front lines were the only organizational contacts available to women. In response, agencies in many cases focused on their workers. This involved supporting the frontline workforce through morale boosting events and changing working conditions.

*…it’s pretty laborious, for them [staff] to be like calling every single person and I think more pressure on them to be like, I’m the only person they’re [the clients] talking to right now. … another piece is …staff fatigue and burnout … COVIDs been really hard … this Friday, we have a staff morale event…*

Sites also instituted work from home policies and flexible scheduling. This benefited staff and clients alike providing who had the autonomy to schedule appointments at times throughout the day and evening.

With COVID-19 came organizational change. Sites had to re-think their approach to client engagement much of what was previously done in person needed to be done virtually. These changes required staff and clients as well as clients to gain new skills related to virtual work. Sites reported helping clients navigate technology and the process of downloading apps needed to support virtual appointments. One site instituted a monthly education series to support this. Through this process, staff also needed to adapt their interventions while simultaneously ensuring they were still being delivered as intended.

*I think what’s making the big difference now is how we are checking in. We are checking in a lot more and that’s probably what we always needed to do…*

*…And then of course COVID. And, you know, having to do a lot of our intervention activities virtually. So, that we remain safe, and … the participants remain safe. A lot of the intervention activities that would normally take place in person, how do you translate that into a virtual engagement and not compromise … the validity of the intervention. … of course [we] will be working very closely with you all to try to problem solve and make adaptations … until we will be able to increase our level of engagement in person.*

### Pandemic relief

Beyond changes to workforce policies, federal and state level expenditures and changes associated with pandemic relief increased access to treatment for Black women. During COVID-19 reimbursement for telehealth services increased and organizations began to develop new protocols for the use of electronic communication with clients, which facilitated access for Black women.

*So, that’s one, a couple things have happened due to COVID-19 that are going to be really helpful for this program… We have the ability for individuals to sign documents for care services, you know, without being in person. We have really perfected a lot about how we do intakes virtually and working with women. … these things that have happened because of COVID-19 are really going to assist us in meeting this hidden population.*

*… COVID-19 really kind of instituted our ability …to write in the IM strategies for using mobile phones to assist [women] ...*

Sites also described the benefits of changes in government policies also increased access to medication for opioid use disorder (MOUD).

*…they changed the rules of the game so that we could deliver more Suboxone to people at home. So, our pharmacies doing that because we want people to [not] go without meds…*

Pandemic assistance programs and grants allowed sites to expand resources to address the social determinants of health.

*Our Care Act money was spent [on] more food and PPE, so we were delivering food and PPE if patients needed masks and stuff like…*

*…with the implications of COVID comes housing and being able to assist with…rental assistance. So that is something that across all of our programs we are focusing on that we have specialized funding to be able to assist with housing.*

These grants included additional funds that allowed sites to provide clients with food, housing, and personal protective equipment (PPE) resources, but more importantly shifts in policies and procedures allowed them to provide services in a meaningful way for women, which in some cases involved expanding services. Sites found that the government was investing material goods and services that Black women needed all along.

## Discussion

Our findings illustrate how COVID-19 exacerbated existing structural barriers impacting Black women with HIV. COVID-19, coupled with existing inequities, has taken a toll on Black women with HIV as well as the staff teams that serve them, many who are also Black women. Like others, we found socioeconomic stressors present under racial capitalism prior to the pandemic have been amplified [28, 29]. Consistent with the literature, Black women with HIV served by demonstration sites have seen increases in unemployment throughout the pandemic [30]. Sites reported women are employed by sectors experiencing job loss, such as the service sector, which is of continued concern as experts predict that job loss coupled with persistent economic exploitation may spur a significant increase in homelessness in the U.S [31].

However, a story emerged about how the pandemic and associated expenditures as well as rule changes at the federal and state levels helped to catalyze organizational change making way for staff to provide meaningful outreach, retention strategies and health care for Black women with HIV. In short, the pandemic forced organizations to pivot. State and federal dollars were used to revamp systems and site staff perfected the art of the virtual intake with safeguards for privacy and confidentiality, developed technology training for women and were able institute technology-based interventions (e.g., telehealth, mHealth and eHealth). This was made possible as the result of the expansion of reimbursement for telehealth; it increased health care access and allowed sites to enhance patient engagement, support decision-making and improve communication in the context of COVID-19. Despite sites’ many efforts, however, not all women reconnected with care. As such, there was a need for additional outreach and engagement efforts to ensure linkage and retention in care and meet service needs.

Sites described unanticipated opportunities associated with the COVID-19 response. Because most systems didn’t work in the context of COVID-19, changes were made. Policies were loosened and as a result sites found ways to broaden access to resources and services in a safe way that worked better for Black women with HIV. These changes included work from home policies, case assistance and housing voucher programs, increasing access to web based and telehealth services, and easier access to medications for HIV and opioid use disorder. In addition, sites discussed the benefits of efforts to prevent eviction and increase including, flexible funding for short and longer term stays in hotels and rental assistance to avoid eviction.

Overall, finding indicate Black women with HIV benefited from pandemic relief benefits such as unemployment benefits, but these are now reduced and policies that could have had similar effect, such as paid family and sick leave, have not been passed [32]. One question to explore further is why it took a pandemic to catalyze systems change? For the economy to function in the context of the pandemic systems needed to give and although public health and social benefits resulted, they were secondary benefits of policies designed to keep the economic systems intact.

Under racial capitalism, the wellbeing of the workers is not the priority and there is a racial hierarchy associated with value [19]. This helps to explain why during COVID-Black women were susceptible give their position in the labor market and unprotected, while others gained protections. It also explains the pivot to business as usual, despite the health and social benefits experienced by Black women with HIV. There is a need for public infrastructure that supports the material needs of all people including Black women with HIV. Such efforts are likely to address the structural determinants of inequities in HIV and advance the well-being of Black women. This would involve changes to systems, for example single payer health care, a living wage, social housing and regulating rents as well as access to collective bargaining through passage of the Protecting the Right to Organize Act of 2021. Adopting and implementing paid sick leave that is afforded federal workers is a step that could support women and their families. Currently, only nine states are enacting these laws [32]. These are just a few examples of systems level changes that would advance the well-being of Black women with HIV. The current National HIV/AIDS Strategy recognizes racism as a serious public health threat and supports policies that promote and sustain the health for Black women with HIV and includes indicators to measure social and economic well being of Black women HIV. The policy changes we highlight may support the achievement of goals outlined in the national strategy.

## Conclusions

Racial capitalism threatens public health, harms Black women with HIV (and others), and undermines agencies funded to serve them. Findings highlight that COVID-19 forced virtual pivots in health care and social service delivery. Some of these pivots benefitted Black women with HIV and it’s critical that government funding and policies allow for the continuation of these changes. The Black Women First Initiative is an important step forward in prioritizing Black women’s health, continued efforts are needed.

## Data Availability

The datasets generated and analyzed during the current study are not publicly available due privacy and ethical concerns but are available from the corresponding author on reasonable request.
